# Cultural elements underlying the community health representative – client relationship on Navajo Nation

**DOI:** 10.1186/s12913-016-1956-7

**Published:** 2017-01-09

**Authors:** Vikas Gampa, Casey Smith, Olivia Muskett, Caroline King, Hannah Sehn, Jamy Malone, Cameron Curley, Chris Brown, Mae-Gilene Begay, Sonya Shin, Adrianne Katrina Nelson

**Affiliations:** 1 Community Outreach and Patient Empowerment Program, 210 East Aztec Ave, Gallup, NM 87301 USA; 2 Department of Global Health and Social Medicine, Harvard Medical School, Boston, MA USA; 3 Division of Global Health Equity, Brigham and Women’s Hospital, Boston, MA USA; 4 Partners in Health, Boston, MA USA; 5 Department of Health, Navajo Nation, Window Rock, AZ USA

**Keywords:** Community Health Workers, Community Health Representatives, Navajo Nation, Indigenous, Culture, American Indian

## Abstract

**Background:**

Navajo Nation Community Health Representatives (CHR) are trained community health workers (CHWs) who provide crucial services for patients and families. The success of the CHRs’ interventions depends on the interactions between the CHRs and their clients. This research investigates the culturally specific factors that build and sustain the CHR-client interaction.

**Methods:**

In-depth interviews were conducted with 16 CHRs on Navajo Nation. Interviews were transcribed and coded according to relevant themes. Code summaries were organized into a narrative using grounded theory techniques.

**Results:**

The analysis revealed four findings critical to the development of a CHR-client relationship. Trust is essential to this relationship and provides a basis for providing quality services to the client. The ability to build and maintain trust is defined by tradition and culture. CHRs must be respectful of the diverse traditional and social practices. Lastly, the passing of clients brings together the CHR, the client’s family, and the community.

**Conclusion:**

Understanding the cultural elements of the CHR-client relationship will inform the work of community partners, clinical providers, and other indigenous communities working to strengthen CHR programs and obtain positive health outcomes among marginalized communities.

**Electronic supplementary material:**

The online version of this article (doi:10.1186/s12913-016-1956-7) contains supplementary material, which is available to authorized users.

## Background

Community health workers (CHWs) are trained community members who provide medical services to their clients (on Navajo Nation, the term “client” is used to refer to the beneficiary of the services of the CHW) [1, 2]. CHWs are currently employed in marginalized communities worldwide to improve health [3, 4]. CHWs are important members of their communities, often becoming the sole medical representatives.

Data regarding CHW programs have demonstrated improved health outcomes and efficacy of culturally-competent trainings [5–7]. Indigenous communities have experienced improved health outcomes through CHW involvement [8, 9]. Prior data focus on the factors that lead to such outcomes and CHWs’ motivations for working in resource-poor settings, but little is understood about the cultural aspects of the interactions between CHWs and their clients [5–7]. The importance of cultural factors to the doctor-patient relationship is well-documented, but few papers discuss such factors for community health workers and their clients [10, 11].

The objective of our research is to investigate and understand the cultural factors that impact the CHW-client relationship, focusing specifically on the Navajo cultural context. These cultural factors are investigated through one-on-one qualitative interviews with CHWs as a case study to understand the importance of culture and the role culture plays in improving health outcomes.

The Navajo Nation Department of Health (NNDOH) oversees the Navajo Nation Community Health Representative & Outreach Program that employs community health workers known as Community Health Representatives (CHRs) [12]. For the purpose of this paper, the term “CHR” will be used to describe Community Health Representatives on Navajo and “CHW” will refer to community health workers or similar roles elsewhere.

CHRs are community members trained to provide health education, conduct health screenings, conduct home safety assessments, and assist with connecting their Navajo clients to important medical, housing, and economic resources. The Navajo Nation CHR Program bridges the gap between providers of Western medicine and community members who often espouse unique diverse cultural values. Understanding the unique cultural factors of each community can impact CHW training and appropriate resource allocation for CHWs in underserved areas to ensure improved health outcomes among the populations that CHWs serve [12].

## Methods

### Location

The Navajo Nation is the largest sovereign nation indigenous to the United States, encompassing parts of Utah, New Mexico, and Arizona. The Navajo Area Indian Health Service operates 6 hospitals, 7 health centers, and 15 clinics. Healthcare systems are regionalized into eight service units, each with at least one clinical facility as well as a team of CHRs, varying from two to fifteen, who provide services to the communities within each service unit [13]. Each CHR is assigned to a specific community (termed Chapter), which has its own local governance. Chapters vary tremendously in size—from hundreds of individuals to thousands of individuals. The number of CHRs in a chapter is determined by the population of the chapter and the number of clients who need close monitoring. Some CHRs are assigned to more than one Chapter, depending on the size of the Chapters.

### Overview of the Navajo Nation Community Health Representative Program

Approximately 100 CHRs are currently employed by the Navajo Nation Department of Health (NNDOH). CHRs are selected by the Navajo Nation Community Health Representative Outreach Program based on applicant experience, skills, and interest in community health. CHRs must be certified nursing assistants (CNAs), at least 18 years of age, and bilingual in Navajo and English. Most CHRs live and work in the Chapter that they serve. Currently, most CHRs are female, although there are no specific requirements with regards to religion, sex, gender, or marital status. Once hired, CHRs receive initial and then ongoing monthly training on prevalent health topics; the NNDOH also covers tuition fees for CHRs to attend classes and receive a Certification in Public Health. While optional, CHRs can apply these credits toward an Associates Degree.

Each CHR is assigned to one or more Chapters, where they serve as a nexus between clinic and community care. CHRs identify clients within their community who are in need of services and may also receive client referrals from clinic-based providers. High-risk patients are typically individuals with chronic health conditions, such as diabetes or heart disease, or social or geographic isolation. While CHRs may have a high-risk caseload of 50–150 patients, they are also responsible for general health promotion for the entire Chapter population, typically 1000–2500 residents. As CNAs, CHRs are certified to monitor vital signs and measure blood sugars using the finger-stick blood glucose method. They are not certified to perform venipuncture or administer injections. CHRs deliver health education, support activities of daily living, and connect clients to resources (doctors, nurses, police, home repairs, transportation services, or other community supports). They also conduct community-level screenings (such as blood pressure checks at health fairs) and provide updates on emerging health issues (such as Rocky Mountain Spotted Fever) at community meetings.

### Sampling

For the purpose of this study, we used purposive sampling, particularly, criterion sampling. Following are the criteria used to identify CHRs [14]. CHRs were identified based on a representative sample drawn from two personal characteristics including the number of years an individual served as a CHR (short and long time defined as > and < 5 years as CHR), and level of CHR collaboration with Chapters (identified by the CHR supervisor). Research staff from the COPE program, a non-profit organization focused on improving health outcomes for native communities, identified CHRs based on years of service. CHR supervisors identified CHRs based on the CHR’s collaboration with local Chapter officials. Each CHR had at least one client enrolled in the COPE program. CHRs were identified from across all eight Service Units. A total of 16 CHRs were interviewed in order to reach thematic saturation. Of the 18 CHRs approached for interview, two declined due to time constraints. CHR representation reflected 7 of the 8 Service Units in the Navajo Area Indian Health Service.

### Data collection and analysis

Individual interviews were conducted using semi-structured interview guides developed by the research staff. Interviews focused on CHRs’ view of their work, their motivations for working as CHRs, their relationships with their clients, and their perspectives on health and wellness, with a particular emphasis on their cultural interpretations. The interview guide was developed with guidance from the Community Health Advisory Panel (CHAP), a community-based group of individuals that guide COPE’s programmatic and research activities on Navajo Nation. Interviews were conducted in private, in the CHRs’ offices by authors VG and CS and lasted between 45 and 90 min. Interviews were transcribed simultaneously because of the limitations in acquiring approval for voice recordings on Navajo Nation. Interviews were conducted until thematic saturation was reached. The transcriptions were recorded with care to maintain accuracy.

The data was analyzed using grounded theory technique [15]. The transcripts were coded throughout the interview process to identify codes and emerging themes. As suggested by researchers well-versed in grounded theory, the process began with open coding of several transcripts. The codes developed from the open coding exercise were used to perform axial coding, during which the codes were organized into categories, followed by selective coding, the phase in which all the codes were organized into categories and a working codebook was developed. The codebook was agreed upon by two Navajo (CS, OM) and two non-Navajo study staff (VG, AKN). Using the online platform Dedoose, all interviews were double coded by VS and CG. All incongruences in coding were discussed and final codes were agreed upon by VS and CG. All coded data were reviewed by two Navajo and two non-Navajo authors. The process of inductive reasoning to reveal all of the main points of the data yielded four interconnected themes that elucidate important elements of the CHR-client relationship.

The study staff identified four main findings and presented the data to the Community Health Advisory Panel (CHAP), whose feedback was incorporated into the analysis (Additional File 1).

## Results

The CHR-client relationship determines a CHR’s ability to connect with the client to successfully achieve positive outcomes. Crucially, the ability to achieve good outcomes depends on the relationships that they form with their clients. Analysis of the transcripts generated four main thematic findings that are important to the development and sustainability of a well-functioning CHR-client relationship.

### Trust plays a vital role in the CHR-client relationship

In this section, we discuss several reasons why trust is important for clients. Further, we discuss specific concerns that clients have in trusting CHRs or other medical professionals. Trust provides a foundation for successful CHR service delivery. Trusting relationships with clients are critical for CHRs to be able to connect and communicate with their clients. Further, such relationships define the quality and delivery of services that are provided. In order to maintain a well-functioning relationship, a CHR must engender trust.

CHRs expressed the importance of practicing sensitivity when discussing the clients’ doubts and concerns. CHRs stated that both respect for boundaries and privacy are important to their clients and that boundaries were expanded once the patient felt more comfortable with the CHR. CHRs often serve their local communities and understand the specific needs and contexts of their clients; this insight allows CHRs to develop community-specific approaches to earn their clients’ trust. Below is a quote by a CHR discussing why trust is important to her client and the specific reason why her client is able to trust her. Specifically, the CHR is referring to the importance of having trust before discussing concerns related to the client’s health and wellbeing.

“So, you know their clan, especially in [name of chapter omitted], because they are just like—I grew up with these people. They are my moms, they are my grandmas or they are my sisters or they are my aunts. I greet them that way in Navajo. Because that way you [can] communicate with them. You are not invading their privacy. You’re just showing that this is a relative coming into their home. If you just go in and start off, I don’t think they like that. You won’t get a positive result.”

Trust is particularly important given the significant history of unethical research practices in indigenous communities [16, 17]. CHRs state that their elderly clients are hesitant to discuss personal health concerns with healthcare providers because of possible negative interactions with the scientific community. One CHR explains her client’s concerns:

“[Elders] say that the doctors, ‘they just come and go, they practice on us.’ You know, that we are just the “guinea pigs.” Something like that I had.”

Without trusting relationships, clients are reluctant to share information. While building trust requires a considerable investment of resources on the part of the CHR, it is necessary for patient comfort and improved patient outcomes. Below is an example of a CHR describing that even though her client may trust her to some to extent, her client may not be entirely truthful with the CHR. The quote below represents the variability in CHR experiences. The CHR recognizes that trust is necessary for the relationship but not sufficient to result in improved outcomes for the patient. She explains that she perseveres in trying to improve her clients’ health.

“You go and see the patient, and she says, “Yeah I’m following the diet.” But her provider says the patient doesn’t and that she rebels, and that she buys the good food but she doesn’t want to eat it. She fries a lot, eats a lot of frybread [food typically made by frying white flour dough in lard], but when you talk to the patient, she tells you a whole different story. So, you know it’s hard to work with someone like her, but I don’t give up though. Now, because of the hyperglycemia, she can’t get her hip replacement done. My goal is to ensure she gets her surgery.”

### Trust is defined and built upon the shared Navajo culture and traditions

In this section, we expound upon the ways in which trust is built and maintained between the CHR and her client. The culturally specific introduction that occurs during the initial home visit contributes to the development of the CHR-client relationship and initiates the process of building trust. CHRs identified the components of a traditional Navajo introduction to include an exchange of Navajo Clans (*K’e*), identification of hometown, and sharing family names. Through the exchange of clans, the CHR and client are able to identify extended familial relationships and roles. Once these relationships and roles are established, they continue to form the bases of the interactions between the CHR and the client, thereby sustaining the trust in the relationship. CHRs indicate that revealing their hometowns and sharing their family backgrounds provide contexts for their clients and define turning points in the clients’ willingness to trust them.

“Everywhere, like if you go into a home and when you introduce yourself [including identifying one’s four clans, one’s parents’ and grandparents’ lineages]—if it’s the first time, you always have to introduce yourself, where you’re from [the chapter to which one belongs] and then that’s when that relationship starts to build up. And it’s like, ‘Oh, okay, you are so-and-so’s granddaughter. She’s related to me like that, so you must be my female relative’—so that relationship starts to build up and, then, with the relationship and the respect there—that's where building of trust starts.”

Disclosure of clans and family history shows respect for the client. CHRs also show respect by addressing clients with affectionate Navajo terms that reinforce familial roles. This quote exemplifies a way in which CHRs are able to maintain the relationship with the client.

“Well, of course, I never greet anyone with [their] name. That’s like a disrespectful thing, you have to greet them like ‘*Nali’* [my grandmother].”

In addition to using Navajo terms of affection, CHRs use the traditional Navajo clanship introduction to connect with clients in meaningful ways to enrich their own work. As CHRs note, the meaningfulness of the work allows them to continue doing it.

“You know I really enjoy my job, because my clients are just not my clients anymore. They are my family, and through clanship all of them are pretty much related to me. And if not, I still refer them to as grandma or grandpa or sister. And that has helped me a lot—the clanship—as we call it *K’e* in Navajo.”

CHRs also demonstrate their use of Navajo language to show respect for tradition and culture, an important part of building trust and working effectively with clients. The Navajo language improves communication especially when clients may not understand disease and wellness terminology in English.

“If I can’t get to them using English, then I go to my Navajo and tell them—I think the Navajo language, you make it your own. For example, I have a girl who is 16. I’m doing a screening and her blood sugar was 300, and she’s like, “So, what happens now?” She was scared and I told her using my Navajo language—bringing her in like she was my daughter—“*shíyazhí*” [daughter]—and they respond to that.”

### CHRs’ ability to connect with clients inherently lies in their unique understanding of, and respect for, Navajo practices and social dynamics

CHRs interact with clients respectfully, using their community-based knowledge and cultural familiarity. CHRs need to use their knowledge of community and culture to be effective. One such characteristic is the proper use of the Navajo language. Another involves spiritual beliefs, given the diverse religious and traditional beliefs on Navajo Nation. Frequently, individuals do not discuss their closely-held religious beliefs and preferences.

“Everyone has their own religion so, as a CHR, I have to respect all religions and that’s one of the places where respect comes out. You have to respect all religions.”

CHRs must navigate the diversity of religious and cultural beliefs carefully, noting that some clients only respond to treatments based in their own belief systems. Depending on the patients’ beliefs and preferences, CHRs often encourage alternative therapies, especially when patients prefer traditional therapies or religious ceremonies.

“She wanted to not take any pill, and she wanted just to rely on tradition—medicine man, [she believes] people have made this happen to her because of land disputes or whatever. And I told her, ‘Go ahead and see your medicine man. Try it, see what happens.’ And she obviously wasn’t getting any better so she finally started taking the medication. So, that was the only time I was a little bit concerned. She was more into getting the medicine man to fix [the illness] and he was giving her all this advice for cure, but it wasn’t working, I think. Yeah, but she came around to taking the pill because it was not getting any better.”

One CHR notes that understanding clients and working with them effectively involves using the Navajo language effectively. The appropriate use of the Navajo language requires skill and an upbringing that emphasizes Navajo culture and values. Navajo culture and values, furthermore, are reflected in the nuances of the language—including correct tone, word choice, and recognizing the power of the tongue to result in actual events [18].

“We always use third person. We never say, ‘This is going to happen to you.’ With third person, it is another thing of being disrespectful—not saying, ‘This is going to happen if you don’t do this,’ like with elders. I guess it’s like a, a terrible spell. You have to say, ‘When people are like this, if they do this, then this will happen.’”

Ultimately, each CHR maintains her clients’ trust through a variety of ways—from respecting and encouraging individuals’ religious and spiritual beliefs that are centered on uniquely Navajo traditions, to understanding and using the Navajo language effectively. Thus, each CHR has unique characteristics, fundamentally shaped by Navajo traditions, that allow for the successful maintenance of trust.

### Loss brings together the CHR, patient’s family, and the community through spirituality and tradition

The loss of a client is a critical moment, and allows the CHR and the family to grieve together. CHRs mention feeling conflicted between expectations to demonstrate leadership and remain “strong,” while experiencing and dealing with their own grief. CHRs often experience loss and develop their own approaches to managing grief. Ultimately, a client’s death helps bring the community together through spirituality, tradition, and prayer [19].

With the passing of their patients, CHRs feel guilt, depression, regret, and sadness.

“Well, personally, for myself, I just take it all in and at some point in time it does catch up with me. And usually I just go off on my own and I just cry or—people see you as being a strong person. They tell you, “Oh, be strong,” stuff like that. But we are only human. So, usually, that’s how I take care of myself and I know that a lot of my coworkers have gone through things like that, with their job and also with their family.”

A CHR’s responsibility to the family and community sometimes results in pressure to maintain a strong public image. Concurrently, as community leaders, CHRs work closely with families to arrange the funeral services.

“A lot of times when there is death in the family, they need a lot of help, things that we can’t do, but we try to help them. Like someone to dig the grave and I try to help them and let them know that. And being there for them. That helps a lot.”

During times of loss, CHRs mention feeling alone and lacking counseling. According to one CHR’s comments, when working in a clinical setting, healthcare professionals can provide support for one another; but “out there,” in the field, where CHRs work, they work individually, and consequently, learn to process the grief individually.

“Well, being in a nursing home, everything is there for you—I’m surrounded by doctors and nurses. When you are out in the field, you are the only one out there to be there for them, and you are by yourself. In a nursing home, you have other CNAs that will help you with the loss.”

Due to the loss that CHRs experience, both as members of the community and as caretakers, CHRs request formalized and structured grief-counseling services to support the grieving process.

“I always—I keep asking. I keep asking for some form of debriefing [for loss].”

While grief counseling is important, CHRs express that working with patients’ families is often a key part of the healing process. CHRs discuss the importance of healing between the CHR and family through traditional spiritual practices and prayer.

“Spiritual…I respect other denominations, the traditional, the Native Americans—I’m in my own community, I know who practices which beliefs, so I respect them and they respect me. I come from a family that is well known for it’s Christianity background, so they know where I am coming from, and I know who’s coming from which camp. We all ask one another for prayers, and, if somebody is sick, or somebody is going through surgery or loss in the family, we usually go there, and encourage them and share our loss with them and donate. That’s how they know us and that’s how we know them too.”

## Discussion

CHWs play an integral role in the medical workforce and are community members that provide the necessary healthcare services to their communities. In order to achieve optimal health outcomes, it is crucial to understand and implement the key features that produce positive outcomes. Studies note training, motivation, and leadership opportunities that can be replicated in numerous settings to produce positive outcomes. These studies fundamentally rely on the community member’s ability to form relationships, the characteristics of which vary according to the community.

**Fig. 1:**
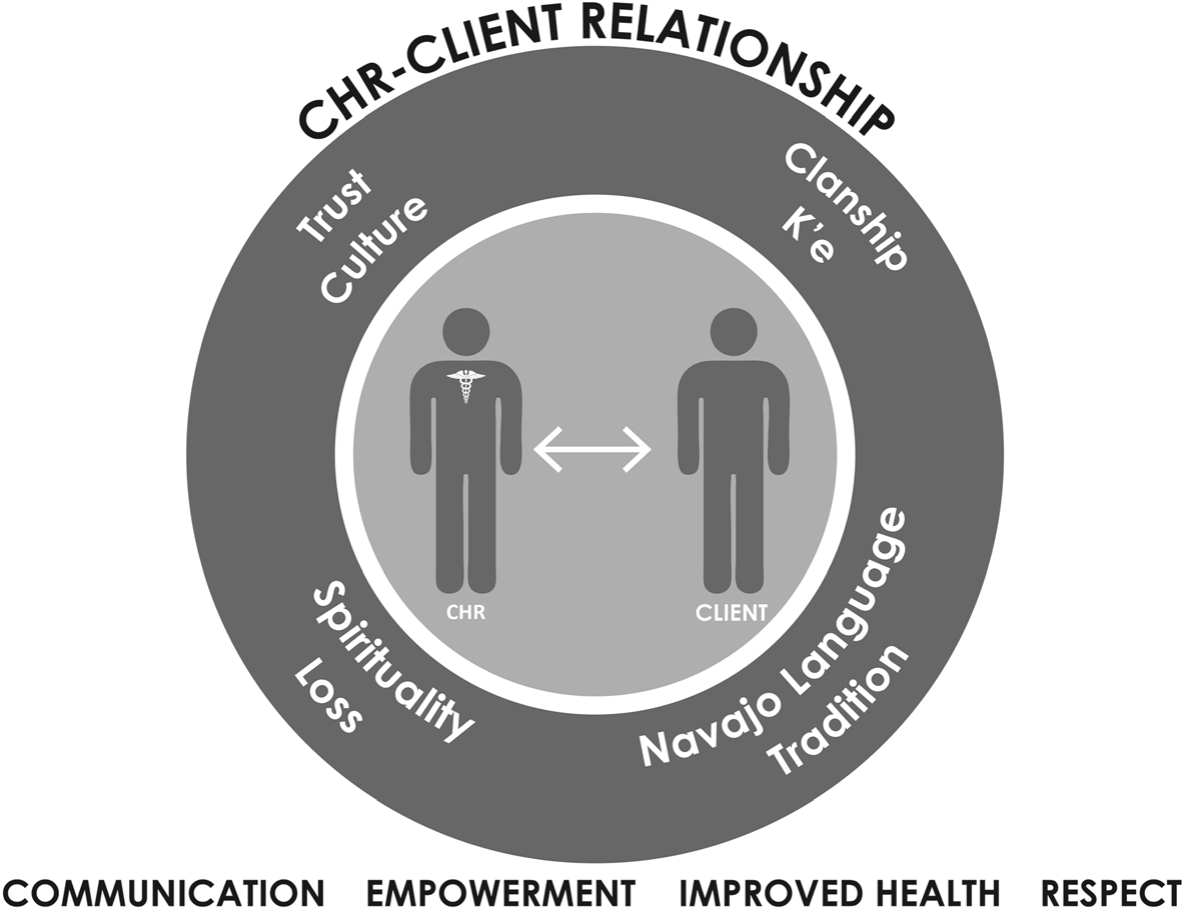
This figure offers a visual description of the relationship between the community health representative and the client. The relationship is sustained and maintained by the factors in the overlying circle. The outcomes of such relationships are listed below: the improvement in communication and health, as well as client empowerment and respect for clients

Although analysis of trust in therapeutic relationships is not novel, we have identified several features of trust related to the Navajo context and culture that ultimately contribute to the formation and deepening of vital relationships between each CHR and her clients (Fig. 1). Trust in the Navajo setting is dependent on historical factors, in addition to the personal factors that are central to relationships in other settings [3–5]. In this paper, we focused primarily on the factors that are important to the individual CHRs. CHRs find that trust is difficult to gain and that clients often do not share the truth. In addition to historical reasons, the client may initially be wary, suspecting that the CHR has been sent by his doctor to “correct” faulty behaviors. However, CHRs build trust through sharing their clans and identifying the familial relationships based on their clans. They further sustain the relationships through their understanding and support of their clients’ unique spiritual practices and traditions, as well as through effective communication in Navajo.

On the Navajo Nation, trust is fundamentally linked to understanding, respecting, and following cultural values. Perhaps the most important aspect of building trust is *k’e* [defined as clanship or kinship], which is defined by an individual’s identity with four of the numerous clan affiliations that are determined through a matrilineal system [20]. The establishment of clans between individuals delineates familial relationships that define the roles and responsibilities of each of the individuals. Sharing this information shows vulnerability on the part of the CHR and engenders confidence among her clients. Additionally, appropriate use of the Navajo language can be difficult, especially for younger CHRs who may have been educated in English-speaking schools, but it is an important element in maintaining a trusting relationship with clients.

CHRs are able to navigate and respect diverse traditional and cultural beliefs. CHRs are “indigenous to the community in which they work—ethnically, linguistically, socioeconomically, experientially” [2]. However, CHRs in large, diverse communities such as Navajo Nation do not necessarily share the experiences and beliefs of their clients. In order to be effective in these communities, CHRs must be understanding of the diversity and must encourage their clients’ beliefs and practices and must refrain from imposing their own beliefs. When clients are disrespected, they may become apprehensive and reluctant to share their own stories.

On Navajo Nation, where the relatively small population is spread over a large area, the elderly clients often live on their own. CHRs are often the first to encounter the passing of clients as the clients’ family members usually do not live with them, and the clients do not always have the means to contact others. Through spiritual and traditional practices that gather and involve the communities, CHRs are able to connect with the clients’ families and engage in healing during these events. CHRs must also learn to cope with grief on their own with limited services to support them through such losses.

We feel that the general themes presented here are relevant to CHWs working in diverse communities. Trust, shared culture, and understanding of the cultural milieu are essential to the CHW-patient relationship. Moreover, a robust relationship is necessary for a CHR’s involvement in an individual’s care and for improved health outcomes.

Our study has several limitations. A qualitative study of 16 interviewees warrants some caution in generalizing the findings to all CHRs. However, the thematic saturation allows us some confidence that these ideas are general to the community and not specific to the CHRs interviewed. Another limitation is our focus on Navajo Nation; however, we find that the general themes have relevance for CHW programs in various cultural contexts. Furthermore, we recognize that our research focuses on the individual factors that impact the CHR-client relationship. As such, a further area of study could focus on the importance of general community perceptions of CHRs and the impact that such perceptions have on the relationships between the CHR and their clients.

## Conclusion

Community health worker programs are crucial to achieving outcomes beneficial to the health of the communities, including improved overall wellbeing for their clients. Culturally sensitive health training for CHWs has been an important aspect in reducing disparities for marginalized communities. However, little has been done to understand the importance of the relationship between the CHW and her client. This study attempts to probe the significant factors of the relationship in a particular setting, with the realization that strengthening such a relationship can have meaningful health benefits for community members. This is particularly important for indigenous communities, including American Indian and Alaskan Native communities.

## Additional file

Additional file 1: CHR Interview Guide. The interview guide was developed by the authors with the help of the Community Health Advisory Panel (CHAP). The general topics covered in the interview guide were discussed during the interviews. (DOCX 90 kb)

## Abbreviations

CHAP: Community Health Advisory Panel
CHR: Community Health Representatives
CHW: Community Health Worker
COPE: Community Outreach and Patient Empowerment Program
NNDOH: Navajo Nation Department of Health
NNHRB: Navajo Nation Human Research Review Board
