# Supplementary material for: Cultural elements underlying the community health representative – client relationship on Navajo Nation
Source: BMC Health Serv Res. 2017 Jan 9;17:19. doi: 10.1186/s12913-016-1956-7 (PMC5223387; doi:10.1186/s12913-016-1956-7)
Supplement: Additional file 1: — CHR Interview Guide. The interview guide was developed by the authors with the help of the Community Health Advisory Panel (CHAP). The general topics covered in the interview guide were discussed during the interviews. (DOCX 90 kb) [file 12913_2016_1956_MOESM1_ESM.docx]

**CHR Interview Guide**

**Background Questions**

Can you tell me a little bit about how you became a CHR? What motivated you to be a part of the CHR program?

What past experience has helped with your work with the CHR Program?

Can you tell me how your ability to speak Navajo has changed since you started working for the CHR Program? Has that changed the way you connect with your clients?

**Work/Actual Home Visits/Interactions with patients**

Tell me about a typical home visit?

How do you explain COPE to a client?

How do you choose which clients to enroll into COPE?

Can you tell me about a time when you felt that you really met the needs of your client? How about a time when it was more difficult to meet their needs?

Is a home visit to a COPE patient different from a home visit to a non-COPE patient?

Have COPE training sessions changed your practice?

**Beliefs + attitudes about role in community/health/family**

What does it mean to be a CHR?

What support does a CHR need in order to be effective?

Can you tell me about how it is working with your chapter?

What does “healthy” mean to you? What does it mean to your clients?

Can you give me an example of your interactions with a client’s family?

How do you deal with loss?
